# Tranquillizing and Thymoleptic Drugs

**Published:** 1965-10

**Authors:** R. E. Hemphill, J. E. Barber

**Affiliations:** Lecturer in Mental Health, University of Bristol, Consultant Psychiatrist, Glenside-Barrow and United Bristol Hospitals; Clinical Research Assistant, Barrow Hospital


### TRANQUILLIZING AND THYMOLEPTIC DRUGS IN OUTPATIENT AND GENERAL PRACTICE

BY

R. E. HEMPHILL, M.A., M.D., D.P.M.

*Lecturer in Mental Health, University of Bristol, Consultant Psychiatrist, Glenside-Barrow and United Bristol Hospitals*

AND

J. E. BARBER, M.B., CH.B., D.(OBST.)R.C.O.G.

**Clinical Research Assistant, Barrow Hospital.*

An account of these drugs was published in this Journal in 1962 (Hemphill, 1962). The present paper is a revision of the earlier survey, and includes a review of the newer drugs and recent developments. For clarity, sections of the original paper have been quoted completely or in part.

The numerous preparations are divided for convenience into tranquillizers, anti-depressants and an intermediate group. This classification is clinical, and does not necessarily imply that the drugs in one group have a similar chemical structure or pharmacological action.

The effect of these drugs in the human is complex, and is not necessarily the same in the sick as in the mentally well. Anxious persons and those with autonomic instability are particularly sensitive to some, and may develop side effects at a low dosage, in contrast to the usual tolerance of schizophrenics and other psychotics.

Since tranquillizers and thymoleptics influence mood and emotion, it is to be expected that they will have an effect on other functions which involve the autonomic nervous system. Control of blood pressure, of intestinal and bladder muscle, and secretion of saliva and alimentary juices, for example, may be interfered with. Hypotension and dry mouth are common side effects, and therapeutic doses of some of the drugs can cause retention of urine and ileus. They should be used with greater care than the older sedatives, and should not be prescribed without a clear reason for the choice of drug and dose. The patient should be warned that prescribed instructions must be followed, that side effects may occur, and that certain foodstuffs, alcohol and other drugs may be forbidden while the tablets are being taken. Relief of symptoms such as agitation, anxiety and insomnia, as well as treatment of the underlying disorder, is particularly important in psychiatry, for this is what the patient will expect. He may be unable to apply himself to the problems of his illness as long as he is disturbed by symptoms, and as long as they are not controlled and are severe he is liable to turn to self-medication. To prescribe tranquillizers as placebos, as is sometimes done, is not justified, and if reassurance only is required, some harmless preparation like Glycero-phosphates or a mild sedative should be given.

The doctor may mistake side effects such as giddiness, hypotension, sleepiness, dulling of initiative, Parkinsonian stiffness or slowing, and involuntary movements of the lips for symptoms of the illness, and increase the dose. This is a serious error, for these may be due to overdosage or idiosyncracy. If they persist when the dose is reduced the patient should be investigated in a psychiatric hospital. It should be noted that continuing or increasing loss of initiative and mental slowing are more likely to

*Working under Research Grant from William R. Warner & Co.

be due to overdosage than a worsening of the psychotic illness. The main principle of treatment should be to arrive at a dose which controls the illness but as far as possible does not produce mental inertia, unpleasant side effects, addiction or dependency, or extrapyramidal symptoms. It is wise from time to time to enquire if the patient is taking any other preparations such as an old prescription, or purchasing "tablets." We have seen many cases where the patient takes other tablets as well as what is prescribed, or even helps himself to a relative's prescription if it has been well spoken of in the family. "I have been taking some of mother's tablets" is a not uncommon remark. The dangers of cheese and other foodstuff, alcohol and amphetamine or dexedrine preparations will be mentioned later, but cannot be stressed too often.

### TRANQUILLIZERS

Tranquillizers reduce mental agitation and turmoil, have a calming effect on restlessness, and in normal doses should not produce sleepiness like barbiturates. Some of them inhibit the production of hallucinations and psychotic thought material from the unconscious, and in this way free the patient from the ensuing disturbances and distractions. This group, of which the large majority, like Chlorpromazine, are phenothiazine derivatives, are indicated in the treatment of schizophrenia.

Some tranquillizers slow down hyperactivity of thought in mania. Others, Librium and Valium for example, have a relaxing and calming effect, but little influence on abnormal thought processes and hallucinations. Stelazine seems to stimulate thought as well as to tranquillize.

There is an intermediate group whose clinical indications are less clearly defined and whose use is more empirical than the other tranquillizers and thymoleptics.

Thus drugs of the tranquillizer and intermediate groups may be given to control symptoms and/or to treat the underlying disorder. They are used to calm agitation, inhibit hallucinations and delusions, reduce hyperactivity in mania, secure relaxation, according to the diagnosis and according to the properties of the particular preparation used.

### ANTI-DEPRESSANTS

Anti-depressants are used in the treatment of clinical depression, and not for the control of symptoms, nor to block hallucinations, nor to reduce manic activity, as are the tranquillizers. It has been found that up to 50 per cent of cases of depression that otherwise would have required E.C.T. will improve within two weeks, and eventually recover when treated with anti-depressants alone. Although a few physicians hold that anti-depressants have no real value in the management of depression, this is obviously untrue. The fact is that since they have been used in general practice, fewer cases of depression are referred for consultant treatment than five years ago. This has been the experience of psychiatrists in hospital and private practice wherever the drugs are widely used.

Thymoleptics may be adjuvants to E.C.T. by reducing the number of electrical treatments required, and thereby the tendency to E.C.T. amnesia; and as maintenance therapy when E.C.T. has produced remission.

Therapy with thymoleptics dates from the observation that Marsilid used in the treatment of tuberculosis often produced elevation of mood and relieved associated depression. It was thought that on theoretical grounds, inhibition of mono-amine-oxidase (an effect Marsilid had on the brain in laboratory conditions) might be a physiological link in the process of the relief of depression. Some of the widely used thymoleptics belong to the group of mono-amine-oxidase (M.A.O.) inhibitors, although there is no proof that the theory is valid in the human. Marsilid is a powerful antidepressant, but most psychiatrists consider it too toxic for general use. M.A.O. inhibitors are possibly less effective in endogenous depression than other thymoleptics, and they are incompatible with some foodstuffs and drugs, but are believed to be useful in the treatment of neurotic forms of depression.

The non-M.A.O. inhibitors, of which Imipramine and Amitriptyline are the most important, have some affinity with Amphetamine. As they are fairly quickly eliminated they may safely be followed by other anti-depressants. They should not be given less than two weeks after stopping an M.A.O. inhibitor because of the risk of cardiovascular disturbances due to summation of effect, as the latter are eliminated slowly.

It should be stressed that only one anti-depressant preparation should be administered to a patient at any one time, and that the patient should be warned that it is dangerous to take any other tablets, and especially anti-depressants, which may have been left over from a previous course of treatment.

### *Mixtures and Combinations*

Capsules and tablets containing mixtures of tranquillizers and anti-depressants in various strengths and combinations are marketed. Combinations of some have been advocated from time to time.

In general, the use of mixtures is to be deprecated, as at the best the dose of one component is usually too small to be effective and a larger dose might be too dangerous. However, combinations containing Stelazine have their adherents, and Parstelin (Parnate + Stelazine) is undoubtedly more effective and more rapid in action than Parnate alone.

The combination of Librium and Nardil is said to be more effective that Nardil alone where depression is accompanied by situational anxiety, but in our experience the addition of even a small dose of Librium to Tofranil can produce alarming postural hypotension. In general, however, with our present limited knowledge of the action and interaction of these drugs, most mixtures should be avoided.

### *Reserpine Derivatives*

With the introduction of the more effective anti-psychotic drugs there is no longer a place for these in psychiatric treatment. Among their disadvantages there is the liability to produce severe depression which does not lift when the drug is discontinued, and is very resistant to any form of treatment.

The trade and approved (pharmacological) names of the majority of preparations on the market, with comments on dosage, side effects and indications, are given in Table I. For convenience, the drugs that are commonly prescribed in the Bristol area head the lists.

TABLE I

Tranquillizers

(P = Phenothiazines)

| *Name* | *Daily Dose* | *Main Use* | *Side Effects* | *Cautions* |
|---|---|---|---|---|
| Largactil (M & B)<br>(Chlorpromazine)<br>P. | 75–1200 mgm<br>(single inj.<br>50–100 mgm) | Psychotic agitation.<br>Schizophrenia. | Skin reactions<br>(i) Photosensitivity<br>(ii) Allergy<br>(iii) Contact dermatitis<br>Parkinsonism<br>Hypotension<br>Tachycardia<br>Polyuria<br>Liver damage. | Prolonged use can produce dyskinesias, occasional blood dyscrasias and deep vein thrombosis.<br>Avoid in/with<br>(i) Hepatic dysfunction<br>(ii) Low leucocyte count<br>(iii) Thiouracil drugs<br>(iv) Hypotensive drugs<br>(v) Recovering alcohol or barbiturate coma. |

Table I—*contd.*

| *Name* | *Daily Dose* | *Main Use* | *Side Effects* | *Cautions* |
|---|---|---|---|---|
| Stelazine (S.K.F.) (Trifluoperazine) P. | 2–30 mgm (Single inj.) 1–2 mgm) | Psychotic agitation. Schizophrenia. | Akathisia Dystonia Parkinsonism | Dyskinesias as with Largactil. Phenothiazines reverse action of Adrenalin |
| Serenace (Searle) (Haloperidol) | 0·75–6 mgm (Single inj. 5 mgm) | Mania. Acute schizophrenia. | Extrapyramidal | Powerful drug safest for hospital use initially. Prescribe with anti-Park. drugs. Phenergan by injection for neurologic crises. Not to be used in arteriosclerosis. |
| Majeptil (Thioproperazine) | (a) 300 mgm (b) 15 mgm (Single inj. 7.5 mgm) | Mania | Opisthotonos Oculogyric crises Sensory loss Autonomic—profuse sweating transient retention Hypotension | As Serenace |
| Fentazin (A & H) (Perphenazine) P. | 6–24 mgm (Single inj. 5 mgm) | Psychotic agitation. Anti-emetic. | Parkinsonism | As Stelazine |
| Sparine (Wyeth) (Promazine) P. | 75–600 mgm (Single inj. 50–100 mgm) | Psychotic agitation | As Largactil but milder. Believed fairly safe in arteriosclerosis and after coronary thrombosis. | Not with low leucocyte count. |
| Tolnate (Prothipendyl HCl) (S.K.F.) | 240–960 mgm | Psychotic agitation. Schizophrenia | Vertigo Headache Dry mouth Postural hypotension. | Incompatible chemically with phenobarb. Not for car drivers. |
| Neulactil (M & B) (Pericyazine) P. | 15–90 mgm (Single inj. 10 mgm) | Psychotic agitation Schizophrenia | Similar to other phenothiazines, side effects milder. | |
| Librium (Roche) (Chlordiazepoxide) | 20–100 mgm | Anxiety Tension Insomnia Alcoholism | Drowsiness Ataxia Constipation Skin rashes | Risk of collapse in elderly |
| Valium (Roche) (Diazepam) | 6–40 mgm | Anxiety Tension Insomnia | Mild ataxia Drowsiness | Ataxia in high dosage Halve dose for elderly |
| Stemetil (M & B) (Prochlorperazine) P. | 15–75 mgm | Psychotic agitation Schizophrenia. | As Largactil | |
| Taractan (Roche) (Chlorprothixine) | 45–300 mgm | Calming epileptic excitement Psychotic agitation | Parkinsonism Hypotension Nasal congestion Paralysis of accommodation Dry mouth | Rapid action potentiates alcohol and narcotics. |
| Dartalan (Searle) (Thiopropazate) P. | 15–30 mgm | Schizophrenia Psychotic agitation Huntingdon's chorea | Extrapyramidal | Avoid barbiturates, alcohol, and in epilepsy. |
| Melleril (Sandoz) (Thioridazine) P. | 75–600 mgm | General tranquillizer Sedative in geriatrics | Hypotension Drowsiness Faintness Photosensitivity Nasal congestion | May cause confusion in elderly |
| Moditen (Squibb) (Fluphenazine) P. | 1–2 mgm mane | Anxiety Tension Schizophrenia | Slight | Avoid if history of epilepsy or fits and with large doses of hypnotics. |

TABLE I—*contd.*

| *Name* | *Daily Dose* | *Main Use* | *Side Effects* | *Cautions* |
|---|---|---|---|---|
| Equanil (Wyeth) Miltown (Lederle) Mepavlon (I.C.I.) (Meprobamate) | 600–1200 mgm | Mild tranquillizer Night sedation (400 mgm) P.M.T. combined with diuretic | Allergy | Contra-indicated (?) in pregnancy. |
| Atarax (Hydroxyzine HCl) | 50–100 mgm | Anxiety Tension Geriatrics | Drowsiness | |
| Notensil (Bayer) (Acetylpromazine) P. | 30–80 mgm | Psychotic agitation Schizophrenia | As Largactil | |
| Pacatal (Warner) (Pecazine) P. | 75–300 mgm | Paranoid schizophrenia | As Largactil | |
| Striatran (N.S.D) (Ethylcamate) | 600 mgm | Anxiety Tension | As Largactil | |
| Vallergan (M & B) (Trimeprazine tartrate) P. | 10–60 mgm | Pruritus associated with nervous tension | Drowsiness Pallor | |
| Veractil (M & B) (Methotrimeprazine) P. | 75–600 mgm | Psychotic agitation Schizophrenia | As Largactil | Not after middle age |
| Vespral (Squibb) (Trifluopromazine) P. | 75–300 mgm | | As Largactil | |

Anti-Depressants (Thymoleptics)

MONO-AMINE-OXYDASE INHIBITORS (see below for cautions common to all)

| *Name* | *Daily Dose (oral)* | *Side Effects* | *Cautions* |
|---|---|---|---|
| Nardil (Warner) (Phenelzine) | 75–150 mgm | Hypotension | Not in liver disease. See table M.A.O.Is. |
| Parnate (S.K.F.) (Tranylcypromine) | 20 mgm | Hypotension Insomnia | As above Last dose before 3 p.m. |
| Parstelin (S.K.F.) (Tranylcypromine+ trifluoperazine) | 20 mgm | Hypotension Insomnia | As above Last dose before 3 p.m. |
| Niamid (Harvey) (Nialamide) | 75–150 mgm | Hypotension | Not in liver disease. Do not give with Chlorothiazide |
| Actomol (I.C.I) (a-methylbenzlhydrazine) | 15–20 mgm | Hypotension ?contraceptive | Avoid in liver and renal disorders and hypotension. Can unmask latent diabetes and cause diabetic coma. Prolonged hypotension may need Noradrenalin drip etc. |
| Drazine (S & N) (Phenoxypropazine) | 10 mgm | Hypotension | Not in liver disease |
| Marplan (Roche) (Isocarboxazid) | 10–30 mgm | As other M.A.I.Os. May be severe. | Toxic reactions |
| Marsilid (Roche) | 150 mgm | As Marplan | Toxic reactions |

THYMOLEPTICS OTHER THAN M.A.O.Is. (should not be prescribed within 14 days after any M.A.O.I.)

| | | | |
|---|---|---|---|
| Laroxyl (Roche) Saroten (Warner) Tryptizol (M.S. & D.) (Amitriptyline) | 30–150 mgm | Drowsiness Nausea Hypotention Fine tremor Headache Heartburn Dry mouth Blurred vision Skin rashes | May precipitate retention, ileus, glaucoma. May activate latent schizophrenia. |

TABLE I—*contd.*

| *Name* | *Daily Dose (oral)* | *Side Effects* | *Cautions* |
|---|---|---|---|
| Tofranil (Geigy) (Imipramine) | 50–150 mgm | Dry mouth<br>Parkinsonism<br>Hypotension | May precipitate retention, ileus, glaucoma. |
| {Allegron (Dista) Aventyl (Lily)} (Nortriptyline) | 30–150 mgm | Anti-cholinergic<br>Dry mouth<br>Drowsiness | As Tofranil |
| Pertofran (Geigy) {n–(8-methylamino propylamino-dibenzyl HCl)} | 50–200 mgm | Sweating<br>Dry mouth<br>Ocular<br>Insomnia | As Tofranil |
| Insidon (Geigy) (Opipramol) | 50–150 mgm | Milder | Mild anti-depressant |

### RESERPINE DERIVATIVES

Rauwolfia — Reserpine (Serpasil) (Sandril) (Reserpex)

Randixin — Deserpidine (Harmonyl)

Tetrabenazine (Nitoman)

*Combinations*

Serpatonil = Reserpine + Ritalin

Carmatin = Reserpine + Mephenesin + Acetylecarbromal

Elimit = Reserpine + Orphenadrine

Neostal = Reserpine + Theophylline + Phenobarb.

Nitensor = Reserpine + 4 barbiturates

### ANTI-PARKINSON DRUGS FOR USE WITH PHENOTHIAZINES

Artane (Lederle) (Benzhexol)

Disipal (Camden) (Orphenadrine)

Congentin (M.S.D.) (Benztropine)

Pipanol (Bayer) (Benzhexol)

Phenergan (M & B) (Promethazine)

Kemadrin (Ward) (Procyclidine)

### COMBINATIONS CONTAINING TRANQUILLIZERS

Amargyl —Largactil + amylobarbitone
Amphactil —Largactil + dexamphetamine
Amylozine —Stelazine + amylobarbitone
Prozine —Sparine + Meprobamate
Parstelin —Stelazine + Parnate
Stelabid —Stelazine + isopropamide
Steladex —Stelazine + dexamphetamine
Tofranil + Promazine—Promazine + imipramine
Triptafen —Fentazine + Amitriptyline

### PREPARATIONS AVAILABLE IN LIQUID FORM

*Tranquillizers*

Fentazin

Largactil

Neulactil

Melleril

Sparine

Stelazine

Stemetil

Vallergan

Valium

*Thymoleptics*

Allegron

Aventyl

Parstelin

Tryptizol

### DRUGS AND FOODS TO BE AVOIDED WITH M.A.O.I.s

*Thymoleptics*

Imipramine

Amitriptyline

Nortriptyline

*Anti-Obesity Agents*

Amphetamine

Appetrol

Barbidex

Dephadren

Desbutal

Dexamed

Dexedrine spans.

Dexten

Durophet

Glandiposam

Levonor

Metamsustac

Steladex

Stimplete

*Others*

Morphine

Pethidine

Adrenalin }*

Ephidrene }

Aldomet

Methidrine

Guanethidine

Reserpine

*Foods*

Mature cheese
Marmite
Bovril
Broad beans
Alcohol

* N.B. Proprietary "cold cures".

### ACUTE PSYCHIATRIC EMERGENCIES

A single injection of the old morphine-hyoscine combination is still the most reliable for settling a wild and excited patient before admission to hospital. The newer drugs are uncertain in speed of action and effect in a single dose, and because of the risk of collapse due to hypotension they should not be used in exhausted, feeble patients, in old age, or with cardiovascular disease. Excitement and psychotic restlessness are usually due to one of the following: acute mania, schizophrenia, alcoholism, brain damage, epilepsy, delirium due to toxaemia or drugs, and cerebral anoxia in pulmonary or cardiovascular disease. For the able-bodied, intramuscular Largactil 50–100 mgm or Stelazine 1–2 mgm, repeated, are effective.

Serenace 5 mgm, and Majeptil 7·5 mgm, which can be repeated, are indicated in acute mania, but the latter should not be given to the ambulant patient for it may produce alarming neurological disturbances including occulogyric crises and opisthotonos, and respiratory distress. Injections of Phenergan (50 mgm) should be given with Serenace and Majeptil to obviate extra-pyramidal trouble; it also increases the sedative effect. Sparine 50–100 mgm by injection can be used safely in senile restlessness. In delirium tremens, Largactil is satisfactory.

All these drugs may potentiate the action of barbiturates and alcohol, and must be used with caution if there is a suspicion that the patient is under the influence of either. Since acute excitement and restlessness may be due to cerebral tumour or head injury, the tranquillizers may mask the diagnosis.

### STATES OF DEPRESSION

These include periodic depression and endogenous depression; depression as a reaction to a stressing event; anxiety or neurotic depression in which a depressive state is associated with anxiety.

About 50 per cent of all cases can be treated with thymoleptics, the remainder will require E.C.T. If the depression is severe or gets rapidly worse, E.C.T. should be given at once, both because of the risk of suicide and because the thymoleptics may not take much effect in less than a week. It is a good rule to consider E.C.T. if there has been no improvement in two weeks, or if after some improvement the case remains stationary after the third week.

There is a wide choice of thymoleptics, and the practitioner should confine himself to a few preparations which he knows well and has found satisfactory.

Imipramine and Amitriptyline are claimed to be more potent and to act more rapidly than the M.A.O. inhibitors. They are quickly eliminated, and they are not incompatible with ordinary foodstuffs, but they readily cause side effects such as hypotension, dry mouth, giddiness and sleepiness. The M.A.O. inhibitors, of which Phenelzine is possibly the best known, are slowly eliminated and they are incompatible with some foodstuffs, but they have been extensively used with safety, and some psychiatrists consider that they are indicated in neurotic and anxiety depression, either alone or prescribed with Librium or Valium.

The usual regime with Imipramine and Amitriptyline is to build up from a small to a high dose, for example 25 mgm b.d. or t.d.s. for three days, and if tolerated then 50 mgm b.d. for one week or longer, increasing to 50 mgm t.d.s. When the patient seems to be well for a month, reduce by two-weekly intervals in the same way.

We believe that no thymoleptics need to be administered continuously for more than three to six months without a break if the patient remains well, and if he does not E.C.T. will be required. Patients may be maintained in a state of mild chronic depression for months or years otherwise. We have seen a patient recently who had been taking an anti-depressant for nearly four years continuously and without any real indications.

In recurrent depression, when the patient has a history of frequent depressive attacks with short intervals, he may be kept well by a small daily dose of a thymoleptic, or a short course of the drugs at intervals, starting about two weeks before the estimated time of a recurrence.

Since drugs may be badly absorbed from the gut in depression, it has been suggested that anti-depressants by injection might act more quickly than by mouth. There is no conclusive proof either way, and because of the risk of hypotension this method is not suitable for outpatient use.

### MANIA

Mania cannot be satisfactorily treated for long outside hospital, but since patients usually resist admission they may have to be treated for a while. Serenace and Majeptil, although very effective, are best reserved for hospital use because of their liability to produce severe side effects, and Chlorpromazine is probably the best substitute.

### SCHIZOPHRENIA

The majority of cases of acute schizophrenia will be treated in hospital. The phenothiazines, including the original Largactil and the more powerful Stelazine, are satisfactory and widely used.

Many cases of schizophrenia, chronic or recent, can be treated as outpatients when a suitable drug and dosage have been arrived at. As these patients are bad witnesses it is important to look for signs of over- or under-dosage. A falling off in work and functional ability and mental dullness indicate overdosage. Hyperactivity, eccentric behaviour, increased delusions and hallucinations indicate increased psychotic mental activity, which calls for an increased dosage. High doses are often used in the acute stages, and these may be reduced according to the previous criteria. Extra-pyramidal disturbances are common accompaniments but occasionally are due to other causes and then will not disappear when the drug has been reduced. Various neurological disorders, including poliomyelitis, may be overlooked in these circumstances.

Prolonged use of phenothiazines may produce prominent dyskinesia first appearing as involuntary movements of the lips and tongue (Evans, 1965; Morphew and Barber, 1965). It is wise to examine patients carefully every three to six months, and to attempt to reduce the dosage. This may not be possible, and we have recently seen a patient, symptom-free for four years, who developed hallucinations immediately his habitual dose of Stelazine was slightly reduced. When phenothiazines are prescribed in high doses for prolonged treatment, anti-Parkinsonian drugs should be taken as well.

### SENILE PATIENTS

Agitation, restlessness and depression are common in senile patients. Where restlessness is only due to boredom and lack of occupation it is wrong to prescribe tranquillizers as this will only lead to mental confusion and toxic effects, but the associated mental unrest common in old people may be helped by tranquillizers. Parkinsonism is easily produced, and hypotensive attacks and collapse may occur. For these reasons the margin of safety of most of the tranquillizers and anti-depressants is narrow.

Sparine is reasonably safe and Melleril is probably the most widely used tranquillizer in geriatrics. It can, however, produce hypotension. Somnolence, confusion and mental vacancy indicate over-dosage. Librium and Valium are helpful at night, but should not be used by day.

In spite of its disadvantages, E.C.T. is probably safer for depression in able-bodied old people than most of the thymoleptics, but claims have been made that Nortriptyline is reasonably safe and effective.

### ANXIETY, WORRY, TENSION AND PHOBIC STATES

Tranquillizers, sedatives and hypnotics are widely prescribed in these conditions. Some tranquillizers certainly help in reducing muscular tension and controlling surges of fear. By controlling symptoms they aid psychotherapy, but are not in themselves a cure, and unless the cause is dealt with the patient is likely to become dependent on the drugs for the relief of fear or insomnia, without getting well. Most patients with anxiety are sensitive to phenothiazines, indeed to all the mood regulators, and readily complain of side effects, giddiness, sweating, blackout and unsteadiness. These in their turn alarm the patients and increase their anxiety. Public announcements about the dangers of some of the drugs, and particularly the risk of cerebral haemorrhage, have greatly reduced their value in anxiety, and sophisticated patients such as nurses and doctors readily develop psychogenic headache and fear when they know they are taking M.A.O. inhibitors. It is a common mistake to increase the dose in order to reduce these symptoms.

For obsessive worry and associated tension Largactil in small doses and Stelazine 2–6 mgm daily are useful. Librium and Valium produce relaxation and are said to relieve muscular tension. For insomnia due to worry, Meprobamate 400 mgm at night is useful. Barbiturates should not be given by day with tranquillizers. Anti-depressants are contra-indicated in anxiety or worry unless a definite diagnosis of depression is made. An attempt should be made to reduce drugs after a couple of weeks, and if there is no improvement in a week or two then the drug is not suitable. Too often patients go on taking drugs because they are afraid to give them up, while admitting that they do them no good. One should not envisage the continuing treatment of anxiety with any of the drugs for more than three months.

There is no confidence-giving drug, and in our opinion Drinamyl and Amphetamine should not be given to inadequate persons or be used in treatment of anxiety. They are very apt to produce dependency and lead to addiction. We have not yet found a drug which will control acute panic and phobic states, but for reducing anxiety before a stressful situation, such as appearing in public, a small dose of any of the tranquillizers mentioned may be helpful. Parstelin seemed to be particularly effective, but in view of certain of its disadvantages it is wiser not to prescribe it for this purpose.

### OBSESSIONAL STATES

Apart from treating an underlying depression, no drugs are effective in obsessional states.

### HYSTERICAL STATES

There is no point in prescribing tranquillizers for hysterics and to do so tends to fix the idea of illness more firmly and obscure the real motivation.

### AGGRESSION AND UNCONTROLLED TEMPER

Before prescribing, an attempt should be made to find the cause, and it is important to exclude outbursts due to temporal lobe epilepsy, and organic brain damage.

For the explosive individual whose outbursts are triggered off by a combination of circumstances, and who "takes it out of" the family and the furniture at home, Valium, Librium and Melleril occasionally help. They may make it easier for the individual to control his temper, and therefore more amenable to psychotherapy. No drug has yet been marketed that will control rage and aggression in humans, as apparently is possible in the laboratory animal.

### ALCOHOLIC ADDICTION

Librium 20 mgm t.d.s. helps to relieve the anxiety and other symptoms when alcohol has been withdrawn, and many patients find it makes life tolerable at this period of treatment.

Chlorpromazine and Stelazine also tranquillize, and the former, as an anti-emetic, quells morning nausea and vomiting. Thymoleptics should not be used unless there is a genuine associated depressive psychosis, and then only if liver function is not impaired.

### EPILEPSY

Epileptics may develop depression or other psychoses. There seems to be no objection to treating them with the appropriate drugs even if they are taking anti-convulsants including phenobarbitone. Although some of the psychotropic drugs such as Librium influence the pattern of E.E.G., their use has been disappointing and they have no place in the treatment of epilepsy. On theoretical grounds Parnate, Tofranil and Largactil should be avoided as they can lower the convulsive threshold.

### BEHAVIOUR DISORDERS IN THE YOUNG

Although Largactil and Stelazine are the most popular, and children are relatively tolerant of them, behaviour disorders of children should not be treated with any drugs before they have been fully investigated in or at hospital.

### PREGNANCY

Tranquillizers and thymoleptics may be used in three general conditions:—

1. For general anxiety and uneasiness in pregnancy.
2. Treatment of a schizophrenic patient who has become pregnant.
3. Depressive states in pregnancy.

There is still insufficient knowledge about the effect of these drugs. The disadvantages to be feared are in connection with the management of the pregnancy and the birth, and the damaging effect on the foetus. It should be possible to avoid their prolonged use in anxiety and uneasiness, but to stop or reduce phenothiazines in a schizophrenic would be to risk a schizophrenic relapse and all that that entails in pregnancy.

Pethedine and Morphine should not be administered along with M.A.O. inhibitors, and the latter must be discontinued before the latter weeks of pregnancy. They are also unsuitable because of the risk of liver damage and their effect on blood pressure.

Largactil and Stelazine have been used for a long time, and there have been no published repor˙s on their teratogenic effect. Largactil, although very effective in hyperemesis, should not be used in prolonged vomiting if there is any suspicion of liver damage.

To summarize, in the present state of knowledge it is wise to avoid the prolonged use of any of these drugs as far as possible. The possibility of teratogenic effects and the effects on labour exists, and we are currently engaged in association with obstetricians in investigating this problem. While the more powerful drugs have not yet been suspected, Meprobamate in the pregnant laboratory rat has led to brain damage or deformity in a significant number of offspring.

### CONDITIONS THAT MAY BE ASSOCIATED WITH PSYCHOTIC AND EMOTIONAL DISTURBANCES

For general guidance Table II summarizes most of these conditions and the use of these drugs according to current practice.

TABLE II

Drugs which have an effect on Associated Conditions

| Condition | Drugs |
|---|---|
| **A. GENERAL MEDICAL** | |
| Arthritis —to reduce worry and ensuing muscular tension | Largactil<br>Librium (acts as specific muscle relaxant)<br>Sparine<br>Tolnate |
| Asthma —to control associated agitation | Largactil<br>Sparine |
| Coronary thrombosis —to control anxiety | Sparine<br>Librium |
| Gastro-intestinal disorders —control of nausea and vomiting in carcinoma; uraemia; radiation contra-indicated with suspicion of obstruction. | Fentazin<br>Largactil<br>Majeptil |
| Genito-Urinary —Noctural enuresis (Munster *et al.*, 1961; Treffert, 1964) | Tofranil<br>Tryptizol |
| —Premature ejaculation | Tofranil<br>M.A.O.I. |
| —Premenstrual tension | Mebrobamate + diuretic |
| Hiccough —especially prolonged from uraemia or coronary thrombosis | Largactil |
| Intractable pain —from carcinoma etc. | Largactil<br>Stelazine<br>Sparine |
| Parkinsonism —with depression (Strang, 1965) | Tofranil (Specific effect in 50 per cent) |
| **B. DERMATOLOGICAL** | |
| Where emotional factors pertain e.g. Eczema, Psoriasis Seborrhoea | Largactil (n.b. may give skin reaction esp. photo-sensitivity)<br>Librium<br>Vallergan (esp. with associated pruritis) |

TABLE II—*contd.*

C. OBSTETRIC

| | |
|---|---|
| Sedation in labour, and to enhance the action of Pethidine etc., also decrease tendency to vomit caused by morphine/pethidine | Fentazin<br>Largactil<br>Sparine |

(N.B. M.A.O.I. must be discontinued in latter weeks of pregancy as they are incompatible with morphia and pethidine)

| | |
|---|---|
| Hyperemesis gravidarum —these drugs should not be used if prolonged vomiting has led to liver failure. | Fentazin<br>Largactil |
| Pre-eclamptic toxaemia | Largactil (esp. in combn. with morphia) |

(N.B. Beware of "false pregnancy" with Largactil as it may cause amenorrhoea, excess wt. gain and false positive frog pregnancy test).

### PRINCIPAL SIDE EFFECTS

Side effects are associated with all psychotropic drugs, especially the most potent therapeutically. The dose at which side effects become troublesome varies from patient to patient. Schizophrenics particularly have a high tolerance. Side effects are most important in the feeble, elderly, and those with cardiovascular disease.

### *Cardiovascular*

At the beginning of treatment, hypotension is common and may produce giddiness, postural blackout and even collapse. It is therefore advisable, in the elderly and susceptible, to confine the patient to bed for the first few days of treatment. Tolerance usually develops rapidly. The M.A.O. inhibitors may cause wide fluctuations in blood pressure if taken with certain foodstuffs such as unprocessed cheese, Marmite, Bovril, broad beans or alcohol, and this can lead to severe occipital headache and on occasion to subarachnoid haemorrhage. Many of the drugs produce transient tachycardia which may cause secondary anxiety. The dose should not be increased in the hope of relieving this.

### *Nervous System*

A common side effect of the phenothiazines is extra-pyramidal Parkinsonism. This calls for a temporary reduction in the dosage. If, as in schizophrenia, high and prolonged dosage is necessary, concommitant anti-Parkinsonian drugs should be prescribed. If Parkinsonism develops in the elderly the drug should be discontinued.

Parnate and Parstelin among others give rise to insomnia, which can be prevented if the second dose is given before 3 p.m. Most of the tranquillizers produce some sleepiness, but if the patient keeps falling asleep during the day or "cannot keep awake", the dose should be reduced.

### *Alimentary*

Dry mouth with difficulty in swallowing are distressing side effects of the non-M.A.O.I. thymoleptics such as Tofranil and Amitriptyline, about which the patient should be warned. This usually becomes tolerable in several days; if not the dose should be reduced. Retention of urine and ileus are rarer complications. The phenothiazines, especially Stelazine, may produce nausea, indigestion and restlessness.

### *Dermatological*

Several of the drugs produce skin rashes, Chlorpromazine perhaps being the worst offender. Besides allergic rashes it can lead to photosensitivity and contact dermatitis.

If photosensitivity develops, ultra violet light barrier cream should be used. Contact dermatitis may occur around the lips when the liquid form is used, or on the hands of nurses and dispensers who handle or crush tablets.

*Jaundice*

Jaundice previously ascribed to the phenothiazines, especially Largactil, is now believed to have been usually due to virus hepatitis, and the real incidence of jaundice is less than 2 per cent. It has been argued that if the jaundice is due to hypersensitivity there will be no permanent liver damage and there is no advantage in discontinuing the drug. Nevertheless, it seems much wiser to discontinue it.

### HAZARDS AND PRECAUTIONS

*Prescribing*

The incidence of suicidal attempts with tranquillizers is increasing, but they are little used for this in comparison with barbiturates, possibly since they do not convey the idea of sleep. It is unwise to give the prescription to a disturbed, confused, or senile patient, and a responsible relative should keep and administer the tablets.

To ensure that psychotropic drug containers are labelled, "Nomen proprium" should be added to the prescription. This is important, as different preparations may be of similar colour. Should the patient be admitted to hospital as a suicide attempt or casualty, and require morphine and its derivatives, it is essential to know what drugs are being taken.

Several of the preparations are in attractive colours and look similar to sweets such as "Smarties". They should, therefore, be kept well away from children. Imipramine, particularly, is a serious poison to young children, even in relatively small doses (Giles, 1963).

Patients must understand the importance of following prescriptions exactly, and not casually as they would do with aspirin. They must not take other tablets as well which they may have hoarded or bought. Much danger could be avoided if patients returned surplus tablets at the end of treatment or when the prescription is changed. Patients rarely know the names of their tablets. They identify them by colour and they even mix indiscriminately tablets of different strength or content because they look alike. As an example, Parnate tablets look similar to ferrous gluconate.

Since the publicity about Thalidomide and cerebral haemorrhage with the M.A.O. inhibitors, patients are apt to be afraid of psychotropic drugs. They may complain of headache, nausea or giddiness after each dose. A placebo may be given, and if the patient continues to complain, the drug is clearly not at fault and this can be explained. Even at the risk of alarming a nervous patient it is an ethical duty of the Practitioner to inform a patient of the possibility of side effects and risks.

The degree of functional ability is the yardstick of improvement in depression and schizophrenia. Increased dullness, lethargy and aimless inactivity are more often due to over- than under-dosage. This can be tested by asking the patient to write a letter or make a little drawing. If there is overdosage the patient may have nothing to say and nothing to draw.

Phenothiazines alone should not be used in the treatment of endogenous depression, but small doses help to control agitation.

Certain thymoleptic drugs may cause Parkinsonism. Unless this is recognized the patient may be treated with heavier doses in the mistaken belief that the depression has deepened.

In anxiety neurosis the initial dose of the tranquillizer should be small and the patient always warned of possible vasomotor disturbances.

*Incompatibility*

Many psychotropic drugs potentiate the action of other drugs and alcohol. This is especially important with the M.A.O. inhibitors, and it is advisable for both practical and legal reasons that all patients on these should carry a card naming the drug and the precautions necessary. These can be obtained from the drug manufacturers. It is unwise to drive a car until tolerance to the drug has been established, and on no account should driving be undertaken while there is any suggestion of giddiness. All car drivers should be urged not to take alcohol while under treatment, and it is not safe to take alcohol until a week after discontinuing thymoleptics.

It is inadvisable to administer psychotropic drugs with hypotensive preparations such as Aldomet, Reserpine and Guanethidine because of potentiation. If it should be necessary, the patient must be under close observation because of the danger of severe hypotensive attacks. Incidentally, these hypotensive drugs may cause aggravation or induction of depression.

*Severe Effects*

Several of the drugs have a marked anti-cholinergic effect, and should therefore not be used in prostatic enlargement with risk of retention. Ileus and glaucoma may also be precipitated.

Blood dyscrasias have been ascribed to the M.A.O. inhibitors and several of the tranquillizers, but this is only to be feared when there is already a low leukocyte count due to other drugs such as Thiouracil derivatives, Chloramphenical, Phenylbutazone or Amidopyrine.

*Obesity*

Depression may accompany obesity and attempts at slimming. Anti-depressants must not be used when anti-obesity pills are being taken because of the Amphetamine or Amphetamine-like substances they may contain. There are at least fourteen such preparations on the market.

*Addiction*

None of the thymoleptics produce dependency or addiction, and in this country only Librium and Meprobamate seem to produce dependency. In some countries where Meprobamate can be bought without medical prescription, it has become a drug of addiction.

From the foregoing it is apparent that psychotropic drugs can lead to serious, even disastrous, reactions, but the risk is slight and need not be a contra-indication provided precautions are taken as with any powerful preparation.

### NEW PREPARATIONS

Since the previous paper (1962) there have been the Thalidomide disasters and the reports of sub-arachnoid haemorrhages when Parstelin and other M.A.O. inhibitors have been taken with cheese.

More recently permanent neurological damage has been described in patients treated with some of the drugs for a long period. These, and the setting up of the Dunlop Committee to control clinical trials and the marketing of new drugs, have rightly introduced great caution into this field. It is now very difficult to carry out satisfactorily clinical trials of preparations not yet released for general use. It seems likely that there will be very few if any additions to the list of tranquillizers and anti-psychotic preparations here reported in the near future.

The opinions expressed in the text and the drugs named do not imply criticism or denigration of other preparations not specifically referred to. They are the result of our own experience and that of our colleagues who have kindly given us their views. No psychiatrist can possibly use more than a few of the drugs available.

The notes in the table are mostly derived from the literature supplied by the manufacturers. To them and to our colleagues we are grateful for their help and advice.

References for the note in the Tables will be found in the memoranda issued by the manufacturers.

### REFERENCES

Evans, J. H. (1965). *Lancet*, **7383**, 458.
Giles, H. McC. (1963). *B.M.J.* **5361**, 844.
Hemphill, R. E. (1962). *Med. J. of the South West.*, **77(i)**, 18.
Morphew, J. A. and Barber, J. E. (1965). *Lancet*, **7386**, 650.
Munster *et al.* (1961). *Amer. J. Psychiat.*, **118**, 76.
Strang, R. R. (1965). *B.M.J.*, **5452**, 33.
Treffert (1964). *Amer. J. Psychiat.*, **121**, 178.

